# Non-Selective PDE4 Inhibition Induces a Rapid and Transient Decrease of Serum Potassium in Mice

**DOI:** 10.3390/biology11111582

**Published:** 2022-10-27

**Authors:** Abigail Boyd, Peter Lochmaier, Daniel Irelan, Edward Fiedler, Ji Young Lee, Brian Fouty, Lina Abou Saleh, Wito Richter

**Affiliations:** 1 Department of Biochemistry & Molecular Biology, College of Medicine, University of South Alabama, Mobile, AL 36688, USA; 2 Center for Lung Biology, College of Medicine, University of South Alabama, Mobile, AL 36688, USA; 3 Department of Internal Medicine, College of Medicine, University of South Alabama, Mobile, AL 36688, USA; 4 Division of Pulmonary and Critical Care Medicine, College of Medicine, University of South Alabama, Mobile, AL 36688, USA; 5 Department of Physiology and Cell Biology, College of Medicine, University of South Alabama, Mobile, AL 36688, USA; 6 Department of Pharmacology, College of Medicine, University of South Alabama, Mobile, AL 36688, USA

**Keywords:** cAMP-phosphodiesterase, PDE4, serum potassium, adrenergic signaling, hypokalemia

## Abstract

**Simple Summary:**

Inhibitors of phosphodiesterase 4 (PDE4), a group of isoenzymes that hydrolyze and inactivate the second messenger cAMP, produce promising therapeutic benefits, including anti-inflammatory and memory-enhancing effects. Here, we report that, unexpectedly, PDE4 inhibitors also reduce serum potassium levels in mice. As both the total potassium content of the body, as well as the distribution of potassium between intra- and extracellular compartments, are critical for normal cellular functions, we further explored this observation. Several structurally distinct PDE4 inhibitors reduce serum potassium levels in mice, suggesting it is a class effect of these drugs. Serum potassium levels decrease within 15 min of drug injection, suggesting that PDE4 inhibition lowers serum potassium levels by promoting a transcellular shift of potassium from the blood into cells. This shift is a characteristically fast process, compared to a loss of total-body potassium via the kidneys or digestive tract (e.g., diarrhea). Indeed, stimulating cAMP synthesis with β-adrenoceptor agonists is known to rapidly shift potassium into cells, and PDE4 inhibitors appear to mimic this process by preventing PDE4-mediated cAMP degradation. Our findings reveal that the various acute physiologic effects of PDE4 inhibitors are paralleled and/or may be affected by reduced serum potassium levels.

**Abstract:**

The analysis of blood samples from mice treated with the PDE4 inhibitor Roflumilast revealed an unexpected reduction in serum potassium levels, while sodium and chloride levels were unaffected. Treatment with several structurally distinct PAN-PDE4 inhibitors, including Roflumilast, Rolipram, RS25344, and YM976 dose-dependently reduced serum potassium levels, indicating the effect is a class-characteristic property. PDE4 inhibition also induces hypothermia and hypokinesia in mice. However, while general anesthesia abrogates these effects of PDE4 inhibitors, potassium levels decrease to similar extents in both awake as well as in fully anesthetized mice. This suggests that the hypokalemic effects of PDE4 inhibitors occur independently of hypothermia and hypokinesia. PDE4 inhibition reduces serum potassium within 15 min of treatment, consistent with a rapid transcellular shift of potassium. Catecholamines promote the uptake of potassium into the cell via increased cAMP signaling. PDE4 appears to modulate these adrenoceptor-mediated effects, as PDE4 inhibition has no additional effects on serum potassium in the presence of saturating doses of the β-adrenoceptor agonist Isoprenaline or the α_2_-blocker Yohimbine, and is partially blocked by pre-treatment with the β-blocker Propranolol. Together, these data suggest that PDE4 inhibitors reduce serum potassium levels by modulating the adrenergic regulation of cellular potassium uptake.

## 1. Introduction

The proper regulation of total-body potassium, and its relative distribution between intra- and extracellular compartments, is critical for normal cellular functions and hence maintained within a narrow homeostatic range [1,2,3,4]. For example, the unequal distribution of potassium across cellular membranes is an important contributor to the membrane potential of cells and, thus, particularly important for the excitability of nerve or muscle cells [1,5,6], with clinically significant hypokalemia (<3.5 meq/L) or hyperkalemia (>5.3 meq/L) predisposing to skeletal muscle dysfunction/weakness and/or cardiac arrhythmias [2]. However, serum potassium levels are still programmed to vary to some degree under normal, physiologic conditions. For example, skeletal muscle contractions, such as during exercise, transiently increase interstitial and serum potassium levels, which may contribute to an initial vasodilation and, hence, increased blood flow supporting muscle function, while also eventually reducing excitability leading to muscle fatigue [1,3,5,7,8].

Total-body potassium levels are principally maintained by adapting to potassium intake and/or unusual potassium loss (e.g., diarrhea) with changes in the rate of potassium reabsorption/excretion by the kidneys—a process that occurs over an hours-long time scale [1,2,3,4]. Indeed, potassium excretion by the kidneys follows a circadian rhythm that matches periods of food- and, hence, potassium intake with increased potassium excretion [2]. Conversely, the re-distribution of potassium between intra- and extracellular compartments occurs on a significantly faster scale, because extracellular (interstitial and serum) potassium levels are sensitive to significant change as they comprise only a small fraction of total-body potassium (~2%), and are much lower than intracellular potassium concentrations [1,2,3,4,5,9]. For example, potassium taken up into the circulation following a meal must be rapidly re-distributed into cells (largely skeletal muscle) to prevent hyperkalemia [9]. Similarly, serum potassium levels are sensitive to potassium release by cell death (e.g., hemolysis, rhabdomyolysis), cellular potassium release via the opening of potassium channels (e.g., exercise), or inhibition of the Na^+^/K^+^-ATPase (e.g., by digitalis glycosides)—the latter being the principal mechanism of transporting potassium into cells [1,10]. Given its critical function, the transcellular shift of potassium into cells is hormonally regulated. Insulin, released following a meal, does not only act to stimulate the uptake of glucose into cells but also promotes cellular potassium uptake by increasing the activity of the Na^+^/K^+^-ATPase [9]. Increased serum catecholamines also promote cellular potassium uptake via β-adrenoceptor-, and, thus, cAMP-dependent mechanisms [1,2,3,5]. Indeed, β-adrenergic agonists, such as Albuterol, are used clinically to rapidly lower potassium levels in hyperkalemic patients [11,12,13,14].

β-adrenoceptors mediate their effects through increasing production of the ubiquitous second messenger cAMP. This increase in the rate of cellular cAMP synthesis is balanced by the rate of cAMP degradation by phosphodiesterases (PDEs), which cleave its cyclic phosphodiester bond to produce 5′-AMP, thus terminating cAMP signaling [15,16,17]. PDEs comprise a superfamily of enzymes that hydrolyze cAMP and the related second messenger cGMP and are grouped into 11 distinct PDE families by sequence homology. The PDE4 family is the largest and comprises four genes or subtypes, PDE4A to PDE4D, that selectively hydrolyze cAMP [18,19,20,21]. PDE4 isoenzymes are widely expressed throughout the body and non-/PAN-selective inhibitors of PDE4 isoenzymes produce numerous therapeutic benefits, including their canonical anti-inflammatory [22,23,24,25], as well as cognition-enhancing [26,27], cardiovascular [28,29], and metabolic [30] effects.

Treatment of mice with PAN-PDE4 inhibitors produces several acute effects, including hypokinesia, hypothermia, and gastroparesis [31,32,33,34]. As part of our efforts to delineate their underlying mechanisms, blood samples from mice treated with the PDE4 inhibitor Roflumilast were subjected to a basic blood chemistry analysis. Roflumilast treatment did not affect serum sodium or chloride levels but induced an unexpected, significant reduction in serum potassium levels. Given the critical impact of serum potassium levels on various cellular and physiologic functions, we further investigated this effect.

## 2. Materials and Methods

### 2.1. Drugs

Rolipram [35] (4-(3-cyclopentyloxy-4-methoxyphenyl)pyrrolidin-2-one), Roflumilast [36,37] (3-(cyclopropylmethoxy)-N-(3,5-dichloropyridin-4-yl)-4-(difluoromethoxy)benzamide), and Yohimbine (methyl (1S,15R,18S,19R,20S)-18-hydroxy-1,3,11,12,14,15,16,17,19,19,20,21-dodecahydroyohimban-19-carboxylate) were from Cayman Chemical (Ann Arbor, MI, USA). YM976 [38,39] (4-(3-chlorophenyl)-1,7-diethylpyrido [2,3-d] pyrimidin-2-one) was from Tocris/Bio-Techne (Minneapolis, MN, USA). Isoprenaline/Isoproterenol (4-[1-hydroxy-2-(propan-2-ylamino)ethyl]benzene-1,2-diol) and Propranolol (1-naphthalen-1-yloxy-3-(propan-2-ylamino)propan-2-ol) were from Millipore Sigma (St. Louis, MO, USA), and RS25344 (1-(3-nitrophenyl)-3-(pyridin-4-ylmethyl) pyrido [2,3-d] pyrimidine-2,4-dione) was obtained from Santa Cruz Biotech (Santa Cruz, CA, USA). All drugs were initially dissolved in DMSO, subsequently diluted into phosphate-buffered saline (PBS), pH 7.4, containing final concentrations of 5% DMSO (dimethyl sulfoxide) and 5% Cremophor EL (MilliporeSigma, St. Louis, MO, USA), and were applied by intraperitoneal (i.p.) injection (100 μL per 20 g body weight).

### 2.2. Animals

Wild-type C57BL/6 mice were generated in-house using breeders obtained from Charles River Laboratories (Wilmington, MA, USA). All mice were maintained in a temperature-controlled (22–23 °C) vivarium with a 12 h light/dark cycle. Animals were group-housed up to four mice per cage and maintained with ad libitum access to food and water. Adult mice ≥ 18 g of body weight, ≥10 weeks of age, and of either sex were used for experimentation by evenly and randomly dividing cage littermates into experimental groups. On the day of an experiment, mice were routinely fasted for 5 h with ad libitum access to water before drug injection and/or experimentation. Experimenters were blinded to the identity of injected drugs until data acquisition and analyses were completed. All experiments and procedures were approved by the University of South Alabama Institutional Animal Care and Use Committee and were conducted in accordance with the guidelines described in the Guide for the Care and Use of Laboratory Animals (National Institutes of Health, Bethesda, MD, USA).

### 2.3. Measurement of Serum Electrolytes

At the end of the specified treatment duration, blood was collected by submandibular bleed under light Isoflurane anesthesia (3% Isoflurane in 1.5 L/min O_2_ for <5 min). The blood was collected into lithium-heparin blood collection tubes (Sarstedt, Nümbrecht, Germany) and subsequently subjected to blood chemistry analysis (Stat Profile Prime+^®^ Critical Care Analyzer, Nova Biomedical, Waltham, MA, USA). Mice were then euthanized using EUTHASOL^®^ euthanasia solution (Patterson Veterinary, Greeley, CO, USA) followed by cervical dislocation. For experiments that were intended to abrogate the effects of PDE4 inhibition on body temperature regulation or locomotion, mice were anesthetized using Isoflurane (3% Isoflurane in 1.5 L/min O_2_) for 10 min before drug injection, and anesthesia was maintained throughout the experiments until after blood samples had been collected.

### 2.4. Measurement of Core Body Temperature

Core body temperature was measured using a thermocouple thermometer (MicroTherma 2T) with a mouse rectal probe (RET-3), both from Braintree Scientific (Braintree, MA, USA), following the manufacturer’s instructions and as reported previously [32,34].

### 2.5. Data and Statistical Analysis

All data are expressed as the mean ± SEM. In scatter dot plots, each dot represents a different animal. For time courses, the n numbers indicate the number of individual animals assessed. GraphPad Prism software (GraphPad Software Inc, San Diego, CA, USA) was used to perform statistical analyses. The Mann–Whitney test with a 95% confidence interval was used to compare two treatment groups and the Kruskal–Wallis test followed by Dunn’s post hoc test were used to determine differences between more than two treatment groups. Experiments comparing the effects of multiple drugs or effects across multiple time points were analyzed using a two-way ANOVA with Sidak’s post hoc test. Statistical differences are indicated as ns (not significant, *p* > 0.05), * (*p* < 0.05), ** (*p* < 0.01), and *** (*p* < 0.001).

## 3. Results

### 3.1. Treatment with the PAN-PDE4 Inhibitor Roflumilast Reduces Serum Potassium, but Not Serum Sodium or Chloride Levels

**Figure 1:**
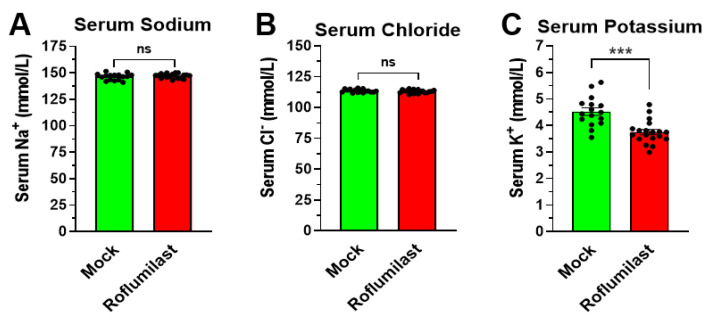
Treatment with the PDE4 inhibitor Roflumilast reduces serum potassium but does not affect sodium or chloride levels. Mice were fasted for 5 h with ad libitum access to water. Animals were then injected with the PAN-PDE4 inhibitor Roflumilast (5 mg/kg, i.p.) or solvent controls (Mock) and blood was collected via cheek bleeds 45 min later. Shown are sodium (**A**), chloride (**B**) and potassium (**C**) levels in the collected blood samples measured using a blood chemistry analyzer. Data represent the mean ± SEM. Statistical significance was determined using Mann–Whitney tests and is indicated as ns (*p* > 0.05) or *** (*p* < 0.001).

**Figure A1:**
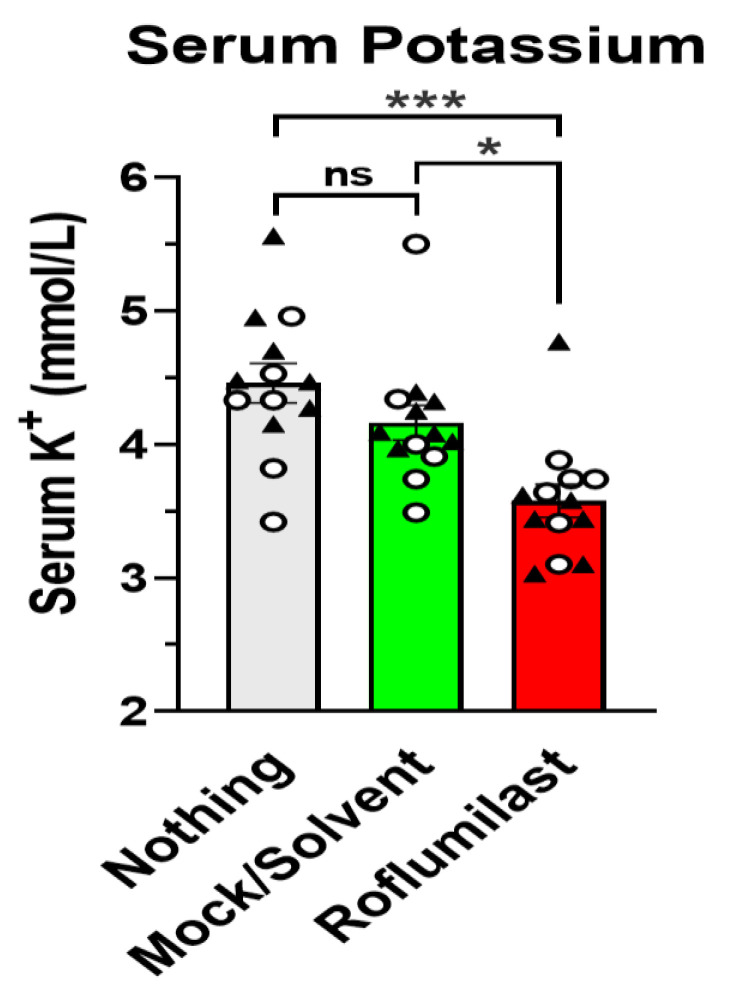
Treatment with the PDE4 inhibitor Roflumilast reduces serum potassium levels. Male (filled triangles) and female (open circles) mice were fasted for 5 h with ad libitum access to water and then divided into three treatment groups: 1. Mice that received no treatment/solution (Nothing), 2. Mice that received an i.p. injection of only the solvent/vehicle, and 3. Mice that received an i.p. injection of the PAN-PDE4 inhibitor Roflumilast (5 mg/kg) dissolved in solvent/vehicle. Blood was collected via cheek bleeds 30 min later and potassium was measured using a blood chemistry analyzer. Data represent the mean ± SEM. Statistical significance was determined using Kruskal–Wallis followed by Dunn’s post hoc test and is indicated as ns (*p* > 0.05), * (*p* < 0.05), and *** (*p* < 0.001).

Treatment with PAN-PDE4 inhibitors, such as Roflumilast, produces several acute physiologic effects in mice, including hypokinesia, hypothermia, and gastroparesis, that reach a maximal amplitude between 30 and 60 min after drug injection [32,33,34,40]. To explore potential underlying mechanisms, blood samples were collected from mice 45 min after treatment with the PDE4 inhibitor Roflumilast for basic blood chemistry analysis. As shown in Figure 1, treatment with Roflumilast did not alter serum sodium levels (Figure 1A) or chloride levels (Figure 1B) compared to solvent controls. Conversely, Roflumilast treatment significantly reduced serum potassium levels (Figure 1C). This hypokalemic effect of Roflumilast is not influenced by the drug vehicle/solvent, which has no effect on serum potassium levels by itself (Appendix A Figure A1), and is not sex specific, with Roflumilast producing comparable reductions in serum potassium in both male and female mice (Appendix A Figure A1). To our knowledge, a role of PDE4 in the regulation of serum potassium levels has not been reported before, which prompted us to explore this finding further.

### 3.2. A Reduction in Serum Potassium Levels Is a Class Effect of PAN-Selective PDE4 Inhibitors

**Figure 2:**
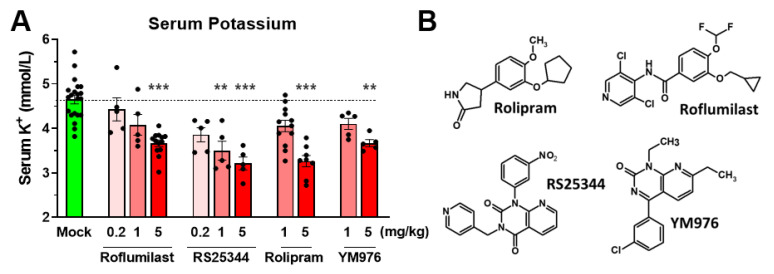
Reducing serum potassium is a class effect of PAN-PDE4 inhibitors. (**A**) Shown are serum potassium levels in mice treated with the indicated doses of the PAN-PDE4 inhibitors Roflumilast, RS25344, Rolipram, and YM976 (0.2, 1, or 5 mg/kg; i.p.) or solvent controls (Mock). (**B**) The chemical structures of the PDE4 inhibitors are shown for comparison. Data represent the mean ± SEM. Statistical significance was determined using Kruskal–Wallis followed by Dunn’s post hoc tests and is indicated as ** (*p* < 0.01), and *** (*p* < 0.001).

To determine whether the reduced potassium levels are an effect unique to Roflumilast, or a class effect of PDE4 inhibition, we treated mice with a number of structurally distinct PAN-PDE4 inhibitors including the archetypal PDE4 inhibitor Rolipram, as well as RS25344 and YM976. As shown in Figure 2, all PDE4 inhibitors reduced serum potassium levels in mice in a dose-dependent manner, suggesting a class effect.

### 3.3. Reduced Serum Potassium Levels Are Not Dependent on PDE4 Inhibitor-Induced Hypothermia or Hypokinesia

**Figure 3:**
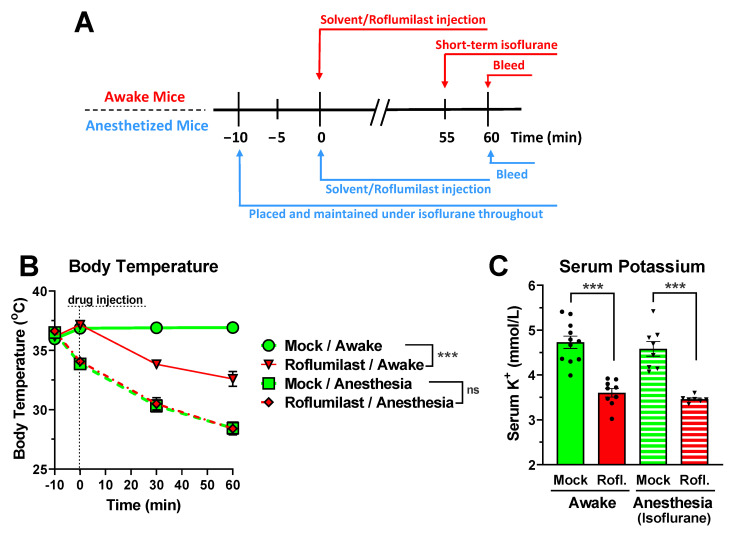
Reduced serum potassium levels are not caused by PDE4 inhibitor-induced hypothermia or hypokinesia. The effect of PDE4 inhibition on serum potassium levels was assessed in awake and anesthetized mice to delineate the impact of acute hypokinesia and hypothermia, which result from PAN-PDE4 inhibition in awake mice, but are abrogated under anesthesia. (**A**) Scheme illustrating experimental steps in assessing the effect of PDE4 inhibition in awake versus anesthetized mice. (**B**) Isoflurane anesthesia abrogates the hypothermic effects of PDE4 inhibition observed in awake mice. The body temperature of mice was measured using a rectal probe at the indicated times prior to and after injection of the PAN-PDE4 inhibitor Roflumilast (5 mg/kg; i.p.; *n* = 8) or solvent control. In awake mice (solid lines), treatment with Roflumilast (red solid line) induces hypothermia compared to solvent controls (green solid line). Conversely, Isoflurane anesthesia started at 10 min prior to PDE4 inhibitor treatment induces hypothermia by itself (green striated line), and injection of the PDE4 inhibitor Roflumilast (red striated line) produces no further effect over solvent controls. (**C**) Comparison of the effect of a 60-min treatment with the PDE4 inhibitor Roflumilast (Rofl.; 5 mg/kg; i.p.) or solvent control (Mock) on serum potassium in awake (solid bars) and anesthetized (Isoflurane, striated bars) mice. Statistical significance was determined using two-way ANOVA with Sidak’s posthoc test and is indicated as ns (*p* > 0.05), and *** (*p* < 0.001).

In humans, as well as in experimental animals, hypothermia is frequently associated with hypokalemia due to an increased transcellular shift of serum potassium into cells [41,42,43,44]. Moreover, as muscle contractions cause a release of potassium from myocytes, differences in the animals’ motility may also affect serum potassium levels [45,46,47]. Thus, we wished to confirm that the observed reduction in serum potassium levels was not simply a consequence of PDE4 inhibitor-induced hypothermia and/or hypokinesia in the animals. General anesthesia efficiently suppresses both central and somatic nervous system functions, thus preventing skeletal muscle contractions. Moreover, anesthesia is well-known to suppress various autonomic nervous system functions, including multiple cold-defense mechanisms (e.g., cutaneous vasoconstriction), leading to hypothermia in both humans and rodents alike [48,49,50,51]. Thus, general anesthesia can efficiently abrogate differences in body temperature and contractile muscle activity between the PDE4 inhibitor and solvent control groups. As shown in Figure 3B, treatment with the PDE4 inhibitor Roflumilast induces hypothermia in conscious/awake mice compared to solvent controls. Conversely, when mice were placed under Isoflurane anesthesia for 10 min before PDE4 inhibitor treatment (see scheme in Figure 3A), there is no longer a difference in body temperature between solvent- and inhibitor-treated mice, given that anesthesia per se induces significant hypothermia (Figure 3B). Importantly, the PDE4 inhibitor Roflumilast produces a similar reduction in serum potassium levels in both awake and anesthetized mice (see scheme in Figure 3C), suggesting that PDE4 inhibitor-induced hypokalemia occurs independent of, and is mechanistically different from, the various nervous system effects associated with PDE4 inhibition in mice [31,32,33,34,40].

### 3.4. The Reduction in Serum Potassium Following PDE4 Inhibitor Treatment Is Rapid in Onset

**Figure 4:**
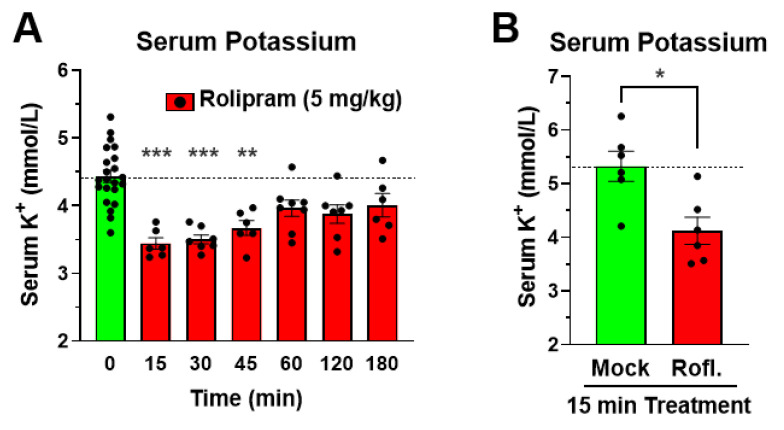
PDE4 inhibition produces a rapid-onset and transient reduction in serum potassium levels. (**A**) Mice were treated with the PDE4 inhibitor Rolipram (5 mg/kg, i.p.) for the indicated time periods before mice were bled to assess serum potassium levels. (**B**) Mice were treated with the PDE4 inhibitor Roflumilast (Rofl.; 5 mg/kg; i.p.) or solvent control (Mock) for 15 min before mice were bled to assess serum potassium levels. All data represent the mean ± SEM. Statistical significance was determined using Mann–Whitney tests (2 groups) or Kruskal–Wallis followed by Dunn’s post hoc tests (more than 2 groups) and is indicated as * (*p* < 0.05), ** (*p* < 0.01), and *** (*p* < 0.001).

As a first step to delineate the mechanism of PDE4 inhibitor-induced reduction in serum potassium levels, we assessed the time-dependency of this effect. As shown in Figure 4A, the reduction in serum potassium upon treatment with the PDE4 inhibitor Rolipram is rapid in onset and already observed at maximal amplitude within 15 min of drug treatment, with potassium levels returning to baseline over a few hours. Similarly, treatment with a distinct PDE4 inhibitor, Roflumilast, also produces a rapid effect at 15 min post drug injection (Figure 4B). This rapid onset suggests that the hypokalemia induced by PAN-selective PDE4 inhibition is likely caused by a transcellular shift of potassium from the vessel lumen into cells, rather than resulting from a loss of serum potassium via the kidneys (reduced reuptake) or digestive system (diarrhea), which occur at a slower pace and are associated with the loss of other electrolytes.

### 3.5. Role of Adrenergic Signaling in Mediating PDE4 Inhibitor-Induced Hypokalemia

**Figure 5:**
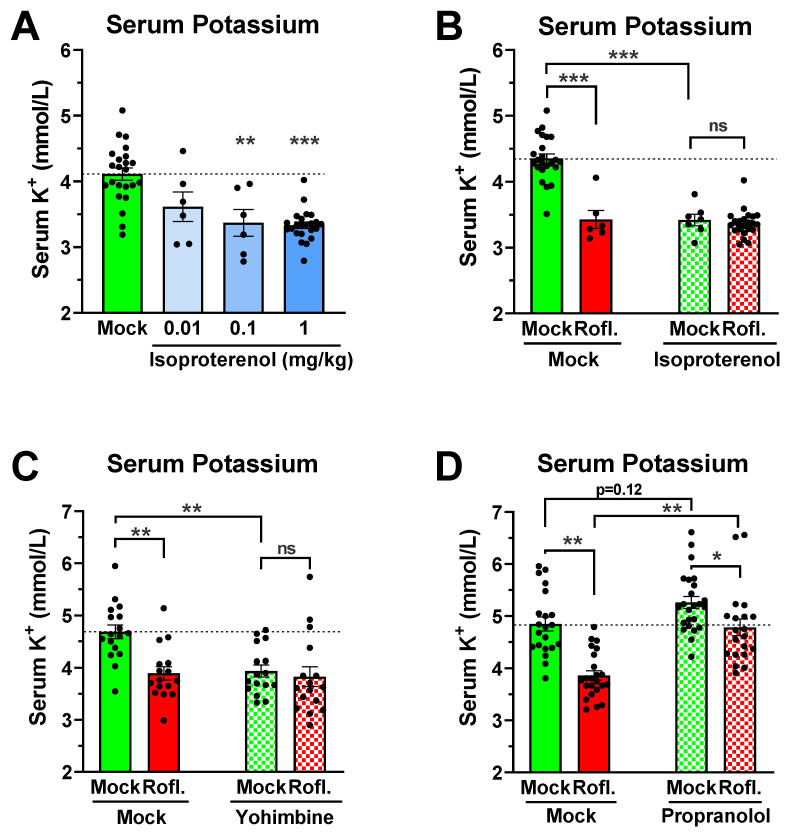
PDE4 inhibitor-induced hypokalemia is mediated by adrenergic receptor signaling. (**A**) β-adrenergic stimulation potently reduces serum potassium levels. Mice were injected (i.p.) with the indicated doses of the β-agonist Isoproterenol and blood was collected 20 min later for assessment of serum potassium levels. (**B**) The effects of Isoproterenol and Roflumilast on serum potassium levels are not additive. Mice were injected (i.p.) with the β-agonist Isoproterenol (1 mg/kg), the PDE4 inhibitor Roflumilast (5 mg/kg), the combination of both drugs, or solvent control (Mock) and blood for assessment of serum potassium levels was collected 20 min later. (**C**,**D**) Adrenoceptor-blockers alleviate the effect of PDE4 inhibition on serum potassium levels. Mice were pre-treated (i.p.) with the α_2_-adrenoceptor blocker Yohimbine (5 mg/kg), the β-blocker Propranolol (5 mg/kg), or solvent controls (Mock), followed 30 min later by treatment with the PDE4 inhibitor Roflumilast (5 mg/kg) or solvent controls (Mock). Blood for serum potassium analysis was drawn at 20 min after Roflumilast/Solvent injection. All data represent the mean ± SEM. Statistical significance was determined using Kruskal–Wallis followed by Dunn’s post hoc test (**A**) or two-way ANOVA with Sidak’s posthoc test (**B**–**D**) and is indicated as ns (*p* > 0.05), * (*p* < 0.05), ** (*p* < 0.01), and *** (*p* < 0.001).

It is well established that increased cAMP signaling, such as upon β-adrenergic stimulation, can rapidly reduce serum potassium levels by shifting potassium into cells (transcellular shift). Indeed, β-agonists, such as Albuterol, are used clinically for the treatment of hyperkalemia [11,12,13,14,52,53]. As this transcellular shift is cAMP-dependent, it may also provide a possible mechanism for the reduction in serum potassium induced by PDE4 inhibitors in mice. As shown in Figure 5A, a 20-min treatment of mice with the β-agonist Isoproterenol potently reduced serum potassium levels, reaching a maximal effect with a dose of 0.1 mg/kg. If PDE4 inhibitors act via the same mechanism, the simultaneous treatment with a PDE4 inhibitor should produce no additive effects in the presence of high doses of Isoproterenol that saturate this cAMP-dependent mechanism of a transcellular potassium shift. In line with this idea, treatment with the PDE4 inhibitor Roflumilast produces no additional effects in the presence of 1 mg/kg Isoproterenol (Figure 5B) compared to treatment with Roflumilast or Isoproterenol alone. Similarly, pretreatment with the α_2_-adrenergic blocker Yohimbine—which increases cAMP signaling by preventing the G_i_-mediated inhibition of adenylyl cyclases—also reduces serum potassium levels [53], and subsequent PDE4 inhibition produces no additional effects (Figure 5C). Finally, pre-treatment with the β-blocker Propranolol produces a non-significant trend toward an increase in serum potassium levels by itself, suggesting that serum potassium levels are regulated by β-adrenoceptor-dependent signaling at baseline [54] (Figure 5D). More importantly, the hypokalemic effect of Roflumilast was reduced by about half following pre-treatment with Propranolol (Figure 5D), a decrease that was statistically significant (*p* < 0.05) using two-way ANOVA. Together, these data support the hypothesis that PDE4 inhibitors lower serum potassium levels, at least in part, by modulating an adrenoceptor-dependent, cAMP-mediated transcellular shift of potassium into cells.

## 4. Discussion

### 4.1. A Reduction in Serum Potassium Levels Is a Class Effect of PAN-PDE4 Inhibitors in Mice

Here, we report that the treatment with PDE4 inhibitors selectively reduces serum potassium levels in mice, whereas serum sodium or chloride levels remain unaffected (Figure 1). Serum potassium levels are dose-dependently reduced by several structurally distinct non-/PAN-selective PDE4 inhibitors, including Roflumilast, RS25344, Rolipram, and YM976, suggesting that this is a class-defining effect of PAN-PDE4 inhibitors (Figure 2). Broad-spectrum PDE inhibitors, such as theophylline or caffeine, have been previously reported to reduce serum potassium in animals [1,12] which, in light of our current findings, may suggest that these drugs reduce serum potassium levels, at least in part, by inhibiting PDE4 enzymes. The PDE4 family comprises four genes or subtypes, PDE4A to D, that are all concurrently inhibited with the commercially available PAN-PDE4 inhibitors tested here. Highly selective inhibitors targeting individual PDE4 subtypes are not yet commercially available. However, the selective knockdown or genetic ablation of individual PDE4 subtypes in cells or animals has clearly established that each may serve unique and non-overlapping physiologic roles [15,55,56]. Thus, which PDE4 subtype, or subtypes, is/are involved in the regulation of serum potassium levels in mice remains to be established in future studies.

### 4.2. A cAMP-Mediated Transcellular Shift of Serum Potassium as a Potential Mechanism of PDE4 Inhibitor-Induced Hypokalemia

Serum potassium levels decrease rapidly but transiently upon i.p. injection of a PDE4 inhibitor, producing a maximal reduction within 15 min, and then slowly recover to normal levels within a few hours (Figure 4). A reduction in serum potassium can arise from many different etiologies, including reduced intake due to poor diet, increased excretion secondary to prolonged emesis or diarrhea, use of diuretics, or kidney disease [57,58,59]. These exert their effects on serum potassium over prolonged periods of time as they gradually lower total-body potassium [59]. Conversely, a shift of serum potassium into cells can occur on a much faster time scale [1,2,3,9]. Treatment with insulin or β-agonists are well established to lower serum potassium levels within minutes and are both used clinically for the treatment of acute hyperkalemia [1,9,11,13,14,57]. Skeletal muscle represents the body’s largest intracellular potassium pool (containing ≥80% of total-body potassium) and is the primary tissue involved in these rapid transcellular shifts [59,60]. At the molecular level, a rapid transcellular potassium shift into cells can be driven by increasing the activity of the Na^+^/K^+^-ATPase, the primary mechanism of cellular potassium uptake [1,5,8,10,61], and/or inhibiting potassium channels (e.g., potassium leak channels) [62], which would otherwise permit potassium to leave the cell. There is sufficient evidence that cAMP levels regulate both potassium transport mechanisms. Increased cAMP/PKA signaling has been shown to directly and/or indirectly stimulate the activity of the Na^+^/K^+^-ATPase [1,10,63], such as via the cAMP/PKA-mediated phosphorylation of phospholemman (also called FXYD1, FXYD Domain Containing Ion Transport Regulator 1), a regulatory/inhibitory subunit of the Na^+^/K^+^-ATPase, that dissociates from the ATPase complex upon PKA-phosphorylation, thereby leading to Na^+^/K^+^-ATPase activation when cellular cAMP levels increase [10,64,65,66]. However, cAMP has also been shown to inhibit potassium leak currents, providing an additional potential mechanism whereby increased cAMP signaling may reduce serum potassium levels [62,67,68,69,70,71,72]. Either mechanism may serve to selectively reduce serum potassium levels without affecting other serum electrolytes, as we observe upon PDE4 inhibitor treatment (Figure 1). For example, activation of the Na^+^/K^+^-ATPase can selectively lower serum potassium (e.g., by 1 to 2 mmol/L) but this would not be expected to significantly increase serum sodium levels, given its negligible addition to the already very high concentration of extracellular sodium under baseline conditions (~145 mmol/L).

The rapid rate with which PDE4 inhibitor treatment lowers serum potassium itself suggests a mechanism that produces a rapid shift of potassium into cells, rather than a loss of total-body potassium. Additionally, it is established that increasing cellular cAMP levels, such as via activation of β-adrenoceptors (particularly β_2_-adrenoceptors) promotes cellular potassium uptake, whereas decreasing cAMP levels, such as via activation of G_i_-coupled α_2_-adrenoceptors, promotes potassium release by tissues [1,2,3,12,53,61,73,74], further suggesting a transcellular mechanism for the hypokalemic effects of PDE4 inhibition. This idea is further confirmed by the observation that PDE4 inhibition produces no additional effects on serum potassium levels in the presence of high doses of the β-agonist Isoproterenol or the α_2_-blocker Yohimbine (Figure 5A–C), suggesting that PDE4 inhibitors act via the same cAMP-dependent signaling mechanisms to induce a potassium shift into cells (e.g., the cAMP-dependent activation of the Na^+^/K^+^-ATPase or inhibition of potassium leak currents). Moreover, when pre-treated with the β-blocker Propranolol, the hypokalemic effect of PDE4 inhibitors is lessened, suggesting that PDE4 acts, at least in part, by controlling subcellular pools of cAMP controlled by β-adrenoceptor signaling.

### 4.3. Potential Physiologic Relevance of PDE4 Inhibitor-Induced Hypokalemia

Treatment of mice with PAN-PDE4 inhibitors induces a vast array of acute effects which span the physiologic landscape: from anti-inflammatory and metabolic effects to distinct responses potentially involving each nervous system, including the central nervous system (improved cognition and memory), the somatic nervous system (hypokinesia), and the autonomic nervous system (hypothermia, reduced gastric motility, salivation), to effects on reproduction and cardiac output [15,19,24,26,28,29,30,75,76,77,78]. Given that PDE4s are widely expressed throughout the body, each of these effects may result from the inactivation of a discrete pool of PDE4 in a single cell type, tissue, or organ. However, as physiologic homeostasis depends on the tight integration of responses from a large number of distinct cells and tissues (e.g., complementary hormonal and neuronal signaling), it is also plausible that some of the observed effects of PDE4 inhibitor treatment are, either directly or indirectly, causally connected. This raises the question of whether any of the established effects of PDE4 inhibitors may cause, or contribute to, the reduction in serum potassium levels we report here. To begin addressing this issue, we explored the potential role of neuronal signaling, given that PDE4 inhibitors produce major nervous system effects. Here, we utilized general anesthesia, as it produces hypnosis and immobility by suppressing neuronal transmission in central and somatic nervous systems, while also ablating many autonomic nervous system responses [48,49,50,51]. For example, as shown in Figure 3B, Isoflurane-induced anesthesia impairs many cold-defense mechanisms, resulting in significant hypothermia. General anesthesia thus abrogates many of the differences between PDE4 inhibitor-treated mice and solvent control groups, including hypokinesia or hypothermia [32,34]. Nevertheless, treatment with PDE4 inhibitors produces similar effects on serum potassium levels in anesthetized and awake mice, suggesting that the PDE4 inhibitor-induced hypokalemia is not caused by changes in neuronal signaling, and supporting the idea of a transcellular shift of potassium caused locally (e.g., amplification of β-adrenergic cAMP signaling within skeletal muscle cells).

Conversely, as a tight regulation of serum potassium levels is particularly critical for the normal function of excitable cells, such as muscle and nerve cells [2,3,5,8,58,79,80], it is quite feasible that the PDE4 inhibitor-induced reduction of serum potassium levels itself may affect muscle function and neuronal signaling in the animals and may modulate some of their reported physiologic effects such as hypokinesia (skeletal muscle), gastric retention (smooth muscle), or nervous system effects such as hypothermia or salivation [31,32,33,34]. Indeed, in multiple experiments, PDE4 inhibitor treatment reduces serum potassium levels in individual animals to at or below 3.5 mmol/L, which represents the lower limit of normal serum potassium levels (homeostatic range is 3.5 to 5.3 mmol/L in humans; [2]). Intriguingly, common presentations associated with hypokalemia in human patients include fatigue [57,59], muscle weakness [57,59], and in some cases hypothermia [41,42]. Thus, future studies may probe the contribution of altered serum potassium levels to the various reported physiologic effects of PDE4 inhibitors.

## 5. Conclusions

Taken together, PAN-selective inhibition of PDE4 enzymes affects a wide array of physiologic paradigms, and exerts many potentially therapeutic benefits, in experimental animals and humans alike [15,19,24,30,75,76,81]. Here, we add yet another, heretofore unrecognized effect of PDE4 inhibitors: a rapid and transient reduction in serum potassium in mice (Figure 1 and Figure 2). Several lines of evidence suggest that the reduction in serum potassium levels induced by PDE4 inhibition results from an acute transcellular shift of potassium into cells, as could be accomplished via activation of the Na^+^/K^+^-ATPase and/or the inhibition of potassium leak channels [2,3,9,10,62]. These include: 1. the rapid onset of PDE4 inhibitor-induced hypokalemia per se (Figure 4); 2. that the hypokalemic effect of PDE4 inhibitors is retained in mice under anesthesia, suggesting it does not occur secondary to established nervous system effects of PDE4 inhibitors and, instead, likely results from increased cAMP signaling in the very cells/tissues that take up the extracellular potassium (Figure 3); and 3. that the effects of PDE4 inhibitors on serum potassium are not additive to saturating doses of cAMP-elevating agents (e.g., β-adrenoceptor agonists and/or α_2_-adrenoceptor antagonists; Figure 5), which are well-known to promote transcellular shifts of serum potassium into cells to lower serum potassium levels [1,2,3]. A proper regulation of serum potassium levels is particularly critical for excitable cells, such as muscle and nerve cells [2,3,5,8,58,79,80]. Thus, it is possible that the PDE4 inhibitor-induced reduction of serum potassium may cause or modulate some of the reported effects of PDE4 inhibitors on smooth- and skeletal muscle function and/or neuronal signaling [31,32,33], which remains to be explored in future studies.

## Data Availability

Not applicable.

## Abbreviations

cAMP: 3′,5′-cyclic adenosine monophosphate; COPD, chronic obstructive pulmonary disease; DMSO, dimethyl sulfoxide; i.p., intraperitoneal; Iso, isoproterenol; PBS, phosphate-buffered saline; PDE, cyclic nucleotide phosphodiesterase; PDE4, cAMP phosphodiesterase 4; PKA, protein kinase A.
